# Impact of psychosocial work factors on risk of medically certified sick leave due to common mental disorders: a nationwide prospective cohort study of Norwegian home care workers

**DOI:** 10.1186/s12889-024-18299-y

**Published:** 2024-03-12

**Authors:** Rigmor Harang Knutsen, Morten Birkeland Nielsen, Lars-Kristian Lunde, Øivind Skare, Håkon A. Johannessen

**Affiliations:** 1 Research Group for Work Psychology and Physiology, National Institute for Occupational Health, Oslo, Norway; 2 Department of Psychology, University of Oslo, Oslo, Norway; 3 Research Group for Occupational Medicine and Epidemiology, National Institute for Occupational Health, Oslo, Norway

**Keywords:** Sick leave, Sickness absence, Home care, Psychosocial work factors, Mental health, Common mental disorders

## Abstract

**Background:**

The Norwegian home care services experience a high level of sick leave, a large proportion of which is due to common mental disorders. A substantial number of such cases can be attributed to psychosocial factors at work, but more knowledge about occupation-specific risk factors is needed to develop targeted preventive measures to reduce sick leave levels. The aim of this study is to identify the most prominent psychosocial work factors influencing the risk of sick leave spells due to common mental disorders.

**Methods:**

Employees from a random sample of 130 Norwegian home care services (*N* = 1.819) completed a baseline survey on 15 psychosocial work factors. Participants were subsequently followed up for 26 months using registry data on sick leave. The outcome measure was the number of medically certified sick leave spells due to common mental disorders during follow-up in the Norwegian social insurance database. Incidence risk ratios (IRR) and 95% confidence intervals (CIs) were calculated using negative binomial regression with robust standard errors.

**Results:**

Emotional dissonance (IRR 1.30, 95% CI 1.05–1.60) and emotional demands (IRR 1.35, 95% CI 1.14–1.58) were associated with an excess risk of sick leave, while control over work pacing (IRR 0.78, 95% CI 0.62–0.98) was associated with a reduced risk. An estimated 30% (95% CI 8.73–48.82) of sick leave cases were attributable to emotional dissonance and 27% (95% CI 4.80-46.33) were attributable to emotional demands. Control over work pacing was estimated to have prevented 20% (95% CI 1.32–37.78) of the sick leave cases.

**Conclusions:**

This study found that emotional dissonance and emotional demands were robust risk factors for sick leave due to common mental disorders, and that control of work pacing constituted a robust protective factor against sick leave.

## Background

Sick leave is associated with significant costs for individuals, employers, and society at large and is known as an important predictor of future disability pension and premature mortality [1, 2]. The amount of sick leave varies substantially between occupations [3, 4], and the female dominated health and social sector displays particularly high levels of long-term sick leave [5]. In Norway, the highest level is found in the home care services (7.7%) where it is nearly twice as high as the average level of the working population (4.3%) [6]. Additionally, both home care services and nursing homes struggle with staff-shortage, high turnover, and recruitment and retainment of nurses [7]. While facing these challenges and a global nurse shortage [8], elder care services must simultaneously adapt to the needs of population ageing due to increased life expectancy and falling fertility rates [9].

Previous studies of the general working population suggest that a significant proportion of all-cause sick leave cases can be attributed to psychosocial risk factors at work [10, 11]. A Norwegian study found that women in the health and social sector were at an increased risk of sick leave compared with women in the general working population [12]. The excess risk was explained by work-related factors, with the most important contributors being psychosocial risk factors. A recent simulation study on hospital employees [13] also demonstrated that improvements in psychosocial work environment had a large potential for reducing onset of sickness absence, particularly long-term. Systematic reviews have found that exposure to psychosocial risk factors is associated with increased risk of mental disorders [14, 15], which is one of the leading causes of sick leave in Norway [5]. The reviews specifically highlight low job control, effort-reward imbalance, low relational and procedural justice, role stress, job strain, high psychological demands, bullying and low social support.

However, results from studies on the general working population or specific sectors may not be generalizable to other sectors, as the magnitude of exposure to risk factors might vary between different professions [16, 17]. Therefore, it is necessary to identify occupation-specific psychosocial risk factors for sick leave in the home care services to support development of effective and targeted preventive measures for reducing sick leave levels.

In the human service sector, there is a paucity of studies that address the relationship between psychosocial stressors and sick leave specifically due to mental disorder. Much of the previous research has been done on self-reported sickness absence data [18–21] or registry data not containing information on underlying diagnosis [22–25]. This indicates a knowledge gap regarding distinct risk factors related to a large part of the sickness absence. We identified six studies using registry data including medically certified diagnostic information on mental disorders. These studies found associations between sickness absence due to mental disorders and job demands [26], role clarity, role conflict, and commitment [27], the combination of high demands and low decision latitude (job strain) [28, 29], social support [28–30], psychological demands, rewards, and effort-reward imbalance [29], and harassment [30]. Finally, Kokkinen, Kouvonen [16] found that human service occupations had a higher risk of sick leave due to mental disorders when compared to other occupations.

Some of the most prominent theoretical approaches to work environment as a determinant of employee health are the job demand-control-support model [31], the effort-reward imbalance model [32], and the organizational justice model [33]. However, some studies suggest that using one model restricts our understanding of the complexity of work environments [18, 34]. A literature review [35] on the effect of psychosocial work factors on long-term sick leave has also called for more research into the role of several factors due to limited evidence; role conflicts, role clarity, low reward, effort-reward imbalance, emotional demands, and indicators of good leadership. Finally, a study on the work environment in the home care sector show that employees may be exposed to several psychosocial risks that are not addressed in the above mentioned models, e.g. role conflicts and emotional demands [36]. Therefore, this study adopted a more comprehensive approach to measuring psychosocial factors by accounting for a broader set of work stressors. This was also recommended by Shahidi, Gignac [34] who found that a comprehensive approach provided greater predictive validity, and Wännström, Peterson [27] suggested that such an approach may be needed to uncover different facets of health issues caused by job stress.

Few studies have previously aimed to elucidate the relationship between the home care work environment and employee health, and we have found no studies on the sector utilizing objective registry data containing diagnostic information. While some studies mentioned above have investigated the association between psychosocial factors and sick leave due to mental disorders in health care workers, there is a paucity of studies examining this association within health care in general and within the home care services in particular. Home care workers are an essential part of municipal health services and increased knowledge of the working conditions specific to this occupation is an important prerequisite for proactive effort to reduce sick leave levels.

Hence, the aim of the present study was to determine the most prominent psychosocial factors influencing sick leave due to mental disorders in the home care services by measuring a broad set of predictors that are proven to be important for health and well-being and by using diagnosis-specific registry data.

## Methods

### Design and study sample

In this prospective cohort study, baseline questionnaire data on working conditions among home care workers were linked to registry data on subsequent sick leave during the 26 months follow-up period. The questionnaire data was collected as part of a project evaluating the effectiveness of the Norwegian Labour Inspection Authority’s regulatory tools for work environment and employee health in the public home care sector [37]. Municipalities and their public home care services were recruited in two waves. To reduce intra-cluster variability and the sample size needed, only municipalities with 20–100 home care workers employed in the public home care services were assessed for eligibility. Additionally, eligible municipalities had not undergone any labor inspection visits in their home care services in the previous year. As of January 2019, Norway comprised 422 municipalities. Out of these, 187 met the eligibility criteria, and 132 were selected at random. In May 2019, 96 municipalities and their public home care services agreed to take part, and invitations were extended to all home care workers within these services, including home care nurses (providing professional medical care) and home care aides (assisting with personal care and housekeeping).

To enhance statistical power for the current study, it was determined that an expansion of home care services was necessary. Eligible municipalities had between 101 and 200 home care workers, totaling *n* = 48. All 48 municipalities were recruited in June 2019, with 34 municipalities agreeing to participate. Invitations were then extended to all home care workers within these municipalities. In total, 130 out of 180 (72%) municipalities consented to participate, and 2591 out of 7103 home care workers agreed to take part (36%). Of these, 1,819 participants approved the linking of survey data to registry data on sick leave, yielding a final response rate of 26%.

### Measures

#### Sickness absence data

The outcome measure of the study was medically certified sick leave spells due to common mental disorders. Complete data on sick leave compensated by the insurance system was provided by the Norwegian Labour and Welfare Administration (NAV). This includes complete registrations of all medically certified sick leave from the first day absent, including the diagnostic codes of the International Classification of Primary Care (ICPC-2) given by the general practitioner. Mental disorder-related sick leave was defined as sickness absence diagnosed within the ICPC category of psychological diagnoses (P) indicating anxiety, depression, and psychological complaints. The following ICPC-2 codes were included in the analysis: *feeling anxious/nervous/tense* (P01), *anxiety disorder/anxiety state* (P74), f*eeling depressed* (P03), *depressive disorder* (P76), *acute stress reaction* (P02), *psychological symptom/complaint/other* (P29). The outcome measure was the number of sick leave spells for each participant with the above-mentioned psychological diagnoses. We obtained sick leave data from March 2019 through June 2021, a follow-up period of 26 months. This data was then linked to the survey data through participants’ unique 11-digit national identity number.

#### Psychosocial working environment instruments

The exposures of the study were work-related psychosocial factors, which were measured using validated scales from the General Nordic Questionnaire for Psychological and Social Factors at Work (QPS^Nordic)^ [27, 38]. The questionnaire was developed to be a comprehensive instrument measuring important psychological and social factors in the workplace with documented relations to well-being and health, and addresses concepts that originate from theories and models on organizational behavior, work motivation, job satisfaction, well-being, job stress and health, such as Job Characteristics Model [39], Job Demand-Control Model [40], Effort-Reward Imbalance Model [41], and several other organizational theories. Furthermore, studies suggest that emotional labor is an important factor in occupations working with clients, such as in the health care sector [42]. This concept is not covered by the QPS^Nordic^ but was included in the study due to its occupational relevance and the above-mentioned call for further research on its impact on sickness absence [35].

The QPS^Nordic^ scales included in the baseline questionnaire were *quantitative demands* (α = 0.8), *decision demands* (α = 0.72), *learning demands* (α = 0.57), *decision control* (α = 0.73), *control over work pacing* (α = 0.79), *role conflict* (α = 0.77), *role clarity* (α = 0.79), *support from the immediate supervisor* (α = 0.89), *empowering leadership* (α = 0.88), *fair leadership* (α = 0.78), *predictability during the next month* (α = 0.59), *human resource primacy* (α = 0.76), *positive challenge at work* (α = 0.74). Each scale contains 3–5 items which measure the frequency of occurrence using the following response alternatives: 1=’very seldom or never’, 2=’seldom’, 3=’sometimes’, 4=’often’, and 5=’very often or always’. Additionally, *emotional dissonance* was measured using four items from the Frankfurt Emotion Work Scales (α = 0.86) [43], and *emotional demands* were measured using one item developed by Statistics Norway [44]: “In your work, to what extent do you need to deal with strong feelings such as sorrow, anger, desperation, frustration, and so on from clients?". Response alternatives were 1=’seldom or never’, 2=’once per week’, 3=’once per day’, 4=’several times per day’, and 5=’several times an hour’.

### Covariates

All analyses were adjusted for self-reported age, sex (male or female), years of education and percentage of full-time equivalent position.

### Statistical analysis

The associations between work-related psychosocial factors and medically certified sick leave spells were examined using negative binomial regression analysis. Sickness absence data is often analyzed using Poisson regression. However, the data is a form of count data that is often characterized by overdispersion, where the variance is larger than the mean [45]. This goes against the Poisson model’s assumption that the variance is equal to the mean. Additionally, the data tends to be zero-inflated (include more values of zero, indicating no sickness absence). To account for this, we used negative binomial regression with robust standard errors to estimate incidence rate ratios (IRR) of the association between exposure and outcome measures. The negative binomial model is a generalization of Poisson regression and better suited to handle overdispersion than the Poisson model. The robust standard errors are robust against unequal distribution of the error terms (heteroscedasticity) and misspecification of the model, and account for multiple testing. All psychosocial factors were analyzed separately with adjustment for covariates. Statistical analyses were performed using STATA 17.0 [46].

Finally, the significant factors were dichotomized at median level (2.2 for all three factors) and analyzed in an additional negative binomial regression analysis. The median split led to responses to the alternatives “very seldom or never” or “seldom” being categorized as low exposure, whereas “sometimes”, “often”, and “very often or always” was categorized as high exposure. To indicate the contribution of the factors to sick leave in the sample, we calculated population attributable risk (PAR) estimates for risk factors and population prevented fraction (PPF) estimates for protective factors. PAR estimates the proportion of cases in the population that can be attributed to a particular exposure. PAR and CIs were calculated as described by Natarajan, Lipsitz [47]. PAR was calculated using the formula (Pd × (IRR– 1/IRR)), where Pd is the proportion of cases exposed to the risk factor (i.e. scores above median) and IRR is the adjusted IRR for sick leave due to common mental disorders. The upper and lower limits of the 95% CI were calculated using the upper and lower limits of the 97.5% CI for Pd and IRR, which represents the 95% Bonferonni CI for PAR.

PPF estimates the proportion of cases in the population that were averted by the presence of a protective factor. PPF was calculated using the formula (Pd(1-IRR))/(1-(1-IRR)(1-Pd)), as recommended by Strain, Brage [48] when potential confounders are present. The upper and lower limits of the 95% CI were calculated by means of simulation as described by Strain and colleagues. We assumed a normal distribution for Pd (mean = 0.517, sd = 0.041) and a normal distribution for ln(IRR) (mean = -0.398, sd = 0.19) and simulated 1 million values of each. From these simulated values, we computed 1 million values of PPF using the formula above. The resulting 1 million estimates were considered to represent the probability distribution of PPF, and the 95% CIs are represented by the 2.5th and 97.5th percentiles of this distribution.

## Results

Characteristics of the study sample are presented in Table 1. The sample consisted mainly of women (95.3%). Mean age was 45.5 (SD 11.86), 48% had a minimum of 13 years of education, 94% were permanently employed, 36% were employed full-time, and 75% had no management responsibilities. About 84% spent a minimum of half their working time with patients. During the 2-year follow-up period, 773 participants (42.5%) experienced at least one period of sick leave due to any cause. Of these, 146 of the participants (18%) experienced sick leave due to common mental disorders. The range for experienced number of sick leave spells due to mental disorders was 1–8, with a median value of 1 (IQR 1–2).

**Table 1:** Characteristics of the study sample

Variables	Total		No medically certified sick leave		Medically certified sick leave due to common mental disorders	
	*N* = 1819	(%)	*N*	(Cases %^1^)	*N*	(Cases %^2^)
**Age**						
< 30	242	13.30	139	57.4	21	8.7
30–39	342	18.80	192	56.1	39	11.4
40–49	477	26.22	270	56.6	50	10.5
50–59	520	28.59	297	57.1	31	6.0
> 59	238	13.08	148	62.2	5	2.1
**Sex**						
Female	1734	95.33	987	56.9	142	8.2
Male	85	4.67	59	69.4	4	4.7
**Work status**						
Permanent employment	1716	94.86	983	57.3	138	8.4
Temporary contract	38	2.10	23	60.5	3	7.9
Substitute/extra	52	2.87	33	63.5	5	9.6
Other	3	0.17	1	33.3	-	-
**Supervisory position**						
Top manager	274	15.32	112	40.9	24	8.8
Middle manager	151	8.44	106	70.2	11	7.3
No management responsibility	1364	76.24	810	59.4	109	8.0
**Working with clients**						
No contact	10	0.55	8	80.0	-	-
Less than half the time	260	14.29	167	64.2	19	7.3
Half the time or more	1532	84.22	862	56.3	127	8.29

^1^ Percentage within each category with no medically certified sick leave

^2^ Percentage within each category with medically certified sick leave due to common mental disorders

Of the 2,591 participants who responded to the baseline questionnaire, 772 participants did not consent to collection of sick leave data during follow-up. T-tests were performed to test for differences in the mean scores on all exposures, covariates and self-reported health measures of this group compared to the 1,819 participants who consented. Results indicated no major differences between the groups.

Means, standard deviations (SD) and the prospective associations between psychosocial factors at baseline and sick leave during follow-up are presented in Table 2. Emotional dissonance (IRR 1.30, 95% CI 1.05–1.60, *p* = 0.03) and emotional demands (IRR 1.35, 95% CI 1.14–1.58, *p* = 0.000) were significantly associated with an excess risk of sick leave due to common mental disorders, whereas control over work pacing was significantly associated with a reduced risk (IRR 0.78, 95% CI 0.62–0.98, *p* = 0.01). The quantitative demands factor presented as significant in the unadjusted model, but this association attenuated to an insignificant level when controlling for covariates. The remaining factors were not significantly associated with sick leave.

**Table 2:** Mean and standard deviations for psychosocial work factors, and incidence risk ratio (IRR) for sick leave due to common mental disorders

Variable	Descriptives		Unadjusted model		Adjusted model^1^	
	Mean	SD	IRR	95% CI	IRR	95% CI
Quantitative demands	3.04	0.78	1.28	(1.03–1.58)*	1.23	(0.94–1.60)
Decision demands	3.63	0.66	1.34	(0.98–1.83)	1.29	(0.95–1.76)
Learning demands	2.56	0.60	1.14	(0.82–1.58)	1.04	(0.72–1.50)
Role clarity	4.30	0.66	0.80	(0.62–1.04)	0.83	(0.62–1.11)
Role conflict	2.68	0.82	1.25	(1.00-1.56)	1.23	(0.96–1.56)
Decision control	2.80	0.71	0.90	(0.69–1.18)	0.89	(0.67–1.18)
Control over work pacing	2.33	0.86	0.79	(0.63-1.00)*	0.78	(0.62–0.98)*
Positive challenge at work	4.21	0.65	1.09	(0.82–1.45)	1.22	(0.92–1.61)
Emotional dissonance	2.36	0.94	1.34	(1.11–1.62)**	1.30	(1.05–1.60)*
Emotional demands	2.70	1.13	1.39	(1.18–1.63)**	1.35	(1.14–1.58)**
Fair leadership	4.02	0.85	1.03	(0.83–1.28)	0.99	(0.78–1.25)
Empowering leadership	3.22	1.05	1.02	(0.85–1.22)	0.98	(0.81–1.18)
Support from immediate supervisor	3.80	1.02	1.04	(0.87–1.24)	1.00	(0.83–1.20)
Human resource primacy	2.98	0.98	1.02	(0.83–1.25)	0.98	(0.79–1.22)
Predictability next month	3.47	0.94	0.92	(0.75–1.14)	0.97	(0.77–1.22)

^1^ Adjusted for age, sex, years of education and percentage of full-time equivalent position

* *P* < 0.05

** *P* < 0.001

The associations between control over work pacing, emotional dissonance, and emotional demands and risk of sick leave spells were robust and persisted in an additional negative binomial regression analysis using factors dichotomized at the median. The excess risk of sick leave associated with emotional dissonance (IRR 1.88, 95% CI 1.26–2.79) and emotional demands (IRR 1.72, 95% CI 1.16–2.55) increased, and the control over work pacing was associated with a further reduction in risk (IRR 0.67, 95% CI 0.46–0.97). The estimated population risk (Table 3) was calculated to quantify the proportion of sick leave due to mental disorder attributable to emotional dissonance, which was estimated at 30% (95% CI 8.73–48.82). The proportion attributable to emotional demands was estimated at 27% (95% CI 4.80-46.33). Finally, the population prevented fraction was calculated to quantify the protective factor’s contribution to avoidance of sick leave. Control over work pacing was estimated to have prevented 20% (95% CI 1.32–37.8) of sick leave due to mental disorder in the sample.

**Table 3:** Population attributable risk (PAR) and population prevented fraction (PPF) of sick leave due to common mental disorders

Variable	*N*	Cases %^1^	IRR (95% CI)	PAR (95% CI)
Emotional dissonance	1776	55.3	1.87 (1.26–2.79)*	30.16% (8.73–48.82)
Emotional demands	1796	54.1	1.72 (1.16–2.55)**	27.07% (4.80-46.33)
				**PPF (95% CI)**
Control over work pacing	1795	55.3	0.67 (0.46–0.97)*	20.22% (1.32–37.78)

* *P* < 0.05

** *P* < 0.01

^1^ Participants with scores above median on the factor

## Discussion

This prospective study aimed to determine the most prominent psychosocial risk and protective factors for sick leave spells due to common mental disorders in a probability sample of Norwegian home care workers. Of the 15 psychosocial work factors investigated, emotional dissonance, emotional demands, and control over work pacing were identified as the most prominent risk and protective factors, respectively. The results showed that 30% of sick leave cases due to common mental disorders were attributable to emotional dissonance, while 27% were attributable to emotional demands. Furthermore, control over work pacing was estimated to have prevented 20% of the sick leave cases.

To the best of our knowledge, this is the first prospective study that addresses psychosocial work factors for sick leave due to common mental disorders among home care workers. Nevertheless, emotional demands are associated with long-term sick leave in longitudinal studies [49] and has been identified as a risk factor for mental disorders in a recent systematic review based on studies of different occupations [50, 51]. Emotional dissonance is found to be associated with mental distress and sickness absence in health and social workers [42]. Hochschild [52] argued that displaying emotions different from your actual felt emotions may lead to detachment from your and other’s emotions and negatively impact mental health over time. Emotional dissonance has also been described as a form of person-role conflict [53], which may explain the link between dissonance and reduced well-being [54]. A significant body of research on emotional labor emphasizes the complexity of emotion regulation and how demanding these processes can be [54–56]. This may require large amounts of individual effort, lead to resource depletion and eventually reduced mental health.

In the QPS_Nordic_ questionnaire, control over work pacing and decision control are facets of job control, which in previous studies have been found to be associated with both sickness absence [57], and absence due to mental disorder [14]. The results from the present study suggest that control over work pacing, and thus being able to take breaks when needed, may protect against sick leave due to mental disorders. Job control has previously been found to buffer against the negative effect of job demands on mental health over time [58, 59]. Furthermore, a recent study by Andersen and colleagues [11] found that scoring favourably on several psychosocial factors could outweigh the negative effects of scoring poorly on one or two other factors. This suggests that while the present study set out to identify the most prominent risk factors for sick leave, the home care services may also benefit from focusing on a wider range of psychosocial factors.

The main strengths of the present study were the prospective study design with a probability sample and the use of diagnosis-specific registry data on medically certified sick leave. There is no risk of common method bias, as the measures of the predictor and outcome variables were obtained from different sources [60]. Due to the use of registry data for the outcome measure, there was no attrition during follow-up and no risk of reporting bias. Additionally, as the sick leave data contains information on diagnoses that was included in the analyses, there was a reduced risk of non-differential measurement errors. While most previous studies of emotional dissonance and risk of sick leave include all diagnoses, the present study advances the understanding of the relationship between psychosocial work factors and sick leave by limiting the analysis to mental disorders. However, the lack of data on previous mental health diagnoses restricts our ability to ascertain whether our findings suggest that emotional dissonance and emotional demands can predict the onset of sickness absence or indicate an increased likelihood of relapse or recurrence in mental health-related absenteeism. The lack of data also restricts our ability to ascertain whether the findings suggest that control over work pacing hinder the onset of sickness absence or indicate a decreased likelihood of relapse or recurrence in mental health-related absenteeism.

To account for heteroscedasticity and multiple testing, we analyzed the data by applying robust standard errors. The results show that several of the predictors were different from 1 in the anticipated direction, although insignificant. Hence, there is a slight risk that we have not detected true effects. On the other hand, there is limited risk that the significant effect of emotional demands and control over work pacing is a result of a Type 1 error.

Despite the use of registry data for determining risk of sickness absence, the study may still be affected by reporting biases as the predictor variables were measured using a self-report questionnaire. The scales used were taken from the QPS^Nordic^, which is a validated measure of psychosocial work factors. It focuses on situation prevalence and consists of items constructed without terms with direct negative or positive connotations to avoid confounding by reporting behaviour [38]. The participants’ responses may not accurately reflect the objective work environment, but their subjective perception of it is argued to be more important [61]. Previous research has applied a variety of approaches to measurement of emotional demands [51, 62] with some focusing on the perception of the demands (e.g. is the work emotionally demanding), and some focusing on assessing their content (e.g. does the work include caring for others’ emotional needs). By measuring the extent to which home care workers need to deal with clients’ strong feelings, the present study relied on content-related emotional demands. This is in line with the focus on situation prevalence in the QPS^Nordic^ items. The study would however have benefitted further from including more items measuring content-related emotional demands to be able to study other aspects of the concept as well.

The sampling strategy is considered to give the study external validity. The results may be largely generalizable to the remaining municipalities’ home care services and possibly other parts of the health and social sector. However, they may not be generalizable to workforces in other national contexts than the Nordic countries due to the uniqueness of their sick leave and disability benefit systems. The final limitation is that the response rate of 26% at baseline is quite low. This introduces the risk of nonresponse bias, though recent research has highlighted that the association between response rates and nonresponse bias is small [63, 64], and that the effects of response rate on the relationships between the variables are likely to be negligible [65]. This is supported by the absence of major differences between participants who consented to collection of sick leave data and those who did not.

## Conclusion

The present prospective study of the Norwegian home care services indicated that psychosocial work factors are associated with medically certified sick leave due to common mental disorders. Specifically, emotional dissonance and emotional demands led to an excess risk of sick leave, whereas control of work pacing led to a reduced risk of sick leave.

The results from this study particularly emphasize the possibility for substantial negative impacts of emotion work and highlights the importance of psychosocial work factors in explaining the increased risk of sick leave in the home care sector. Interventions aiming to reduce medically certified sick leave due to mental disorder may benefit from focusing on management of emotion work, reducing effects of emotional dissonance and emotional demands, and enabling control of work pacing. To achieve this, however, it might be necessary for future research to further investigate how the negative effects of emotional labor factors can be reduced. This includes a better understanding of potential moderator variables that influence the relationship between psychosocial exposures and sickness absence, as well as how a successful intervention should be designed and implemented.

## Data Availability

The data that support the findings of this study are available from Norwegian Agency for Shared Services in Education and Research (SIKT) but restrictions apply to the availability of these data, which were used under license for the current study, and so are not publicly available. Data access request must be submitted to SIKT for review (URL: https://sikt.no/en/home). Contact the Data Protection Officer at the National Institute of Occupational Health, Jan S. Emberland at jan.s.emberland@stami.no.
